# Supplementary material for: In Vivo Intra‐Uterine Delivery of TAT‐Fused Cre Recombinase and CRISPR/Cas9 Editing System in Mice Unveil Histopathology of Pten/p53‐Deficient Endometrial Cancers
Source: Adv Sci (Weinh). 2023 Sep 25;10(32):2303134. doi: 10.1002/advs.202303134 (PMC10646277; doi:10.1002/advs.202303134)
Supplement: Supplementary file 1 — Supporting Information [file ADVS-10-2303134-s001.pdf]

ADVANCED SCIENCE Open Access

Supporting Information

for *Adv. Sci.*, DOI 10.1002/advs.202303134

In Vivo Intra-Uterine Delivery of TAT-Fused Cre Recombinase and CRISPR/Cas9 Editing System in Mice Unveil Histopathology of Pten/p53-Deficient Endometrial Cancers

*Raúl Navaridas, Maria Vidal-Sabanés, Anna Ruiz-Mitjana, Gisela Altés, Aida Perramon-Güell, Andree Yeramian, Joaquim Egea, Mario Encinas, Sonia Gatius, Xavier Matias-Guiu and Xavier Dolcet**

SUPPORTING INFORMATION

***In vivo* intra-uterine delivery of TAT-fused Cre recombinase and CRISPR/Cas9 editing system in mice unveil histopathology of Pten/p53-deficient endometrial cancers.**

Raúl Navaridas[1], Maria Vidal-Sabanés[1], Anna Ruiz-Mitjana[1], Gisela Altés, Aida Perramon-Güell[1], Andree Yeramian[1], Joaquim Egea[1], Mario Encinas[1], Sonia Gatius[2], Xavier Matias-Guiu[2], Xavier Dolcet[1].

[1]Developmental and Oncogenic Signalling Group. Departament de Ciències Mèdiques Bàsiques and Departament de Medicina Experimental. Universitat de Lleida. Institut de Recerca Biomèdica de Lleida, IRBLleida. Lleida, Spain

[2]Oncologic Pathology Group. Departament de Ciències Mèdiques Bàsiques, Universitat de Lleida. Institut de Recerca Biomèdica de Lleida, IRBLleida. Lleida, CIBERONC, Spain.

Supplementary Methods.

Table SM1. Genotyping primers and PCR conditions.

Strain	Primers		PCR protocol			Resulting bands	
			T (ºC)	times	Cycle	Genotype	bsands
**Cre:ER^T**	Fwd<br>Rev	ACG AAC CTG GTC GAA ATC GT GCG<br>CGG TCG ATG CAA CGA GTG ATG AG	94 ºC	2’	1	Cre:ER$^{T-/-}$<br>Cre:ER$^{T+/-}$	no band<br>350 bp
			94 ºC	45’’	32		
			65 ºC	45’’			
			72 ºC	45’’			
			72 ºC	5’	1		
***Pten* floxed**	Fwd<br>Rev	CAA GCA CTC TGC GAA CTG AG<br>AAG TTT TTG AAG GCA AGA TGC	94 ºC	3’	1	PTEN$^{+/+}$<br>PTEN$^{f/+}$<br>PTEN$^{f/f}$	156 bp<br>156 y 328 bp<br>328 bp
			94 ºC	30’’	35		
			60 ºC	1’			
			72 ºC	2’			
			72 ºC	2’	1		
p53 floxed	WT<br>Rev	CAC AAA AAC AGG TTA AAC CCA G<br>AGC ACA TAG GAG GCA GAG AC	94 ºC	3’	1	P53$^{+/+}$<br>P53$^{f/+}$<br>P53$^{f/f}$	288 bp<br>288 y 370 bp<br>370 bp
			94 ºC	30’’	35		
			59 ºC	1’			
			72 ºC	1’			
			72 ºC	3’	1		
mT/mG	Común<br>WT<br>Mutante	CTC TGC TGC CTC CTG GCT TCT<br>CGA GGC GGA TCA CAA GCA ATA<br>TCA ATG GGC GGG GGT CGT T	94 ºC	2’	1	mT/mG$^{+/+}$<br>mT/mG$^{f/+}$<br>mT/mG$^{f/f}$	330 bp<br>250y 330 bp<br>250 bp
			94 ºC	30’’	35		
			57 ºC	1’			
			72 ºC	1’			
			72 ºC	2’	1		

Table SM2. Antibodies used for immunohistochemistry.

Antibody	Dilution	Commertial Company	Catlao¡log	EnVision™ FLEX	Secondary antibody
**PTEN**	1:100	Dako	M3627	*High pH*	EV FLEX Kit
**Ki-67**	1:50	Dako	M7249	*Low pH*	EV FLEX Kit
**ERG**	RTU	Dako	IR659	*High pH*	EV FLEX Kit
**αER (1D5)**	RTU	Dako	IR657	*High pH*	EV FLEX Kit
**E-cadherin**	RTU	Dako	IR059	*High pH*	EV FLEX Kit
**TTF1**	RTU	Dako	M3575	*High pH*	EV FLEX Kit
**Cytoqueratin 8**	1:200	DSHB	AB531826	*High pH*	Rat anti-biotin
**αSMA**	RTU	Dako	IR611	*High pH*	EV FLEX Kit
**h-caldesmon**	RTU	Dako	GA054	*High pH*	EV FLEX Kit
**Calretinin**	RTU	Dako	IR627	*High pH*	EV FLEX Kit
**CD10**	RTU	Dako	GA648	*High pH*	EV FLEX Kit
**Desmin**	RTU	Dako	IR606	*High pH*	EV FLEX Kit
**PAX8**	1:100	GENOVA	AP10903	*High pH*	EV FLEX Kit
**GFP**	1:100	Rockland	600-101-215	*High pH*	Goat anti-biotina
**p-AKT (Ser473)**	1:50	Cell signalling	3787	*High pH*	Rabbit anti-biotin
**EnVision FLEX detection kit**	RTU	Dako	K8002	-	-
**Goat anti-biotin**	1:200	Santacruz	SC-2489	-	-
**Rat anti-biotin**	1:200	ABCAM	AB6733	-	-
**Rabbit anti-biotin**	1:200	Jackson	111-065-144	-	-
**Estreptavidin-HRP**	1:400	Dako	P0397	-	-

Table SM3. Antibodies used in immunofluorescence.

Antigen	Dilution	Commertial company	Catalog
**Phalloidin**	1:1000	Sigma-Aldrich	P1951
**Vimentin**	1:200	BD bioscience	550513
**Cytoqueratin**	1:200	Abcam	9377
**Anti-mouse IgG Alexa Fluor™ 488**	1:250	ThermoFisher	A11029
**Anti-mouse IgG Alexa Fluor™ 546**	1:250	ThermoFisher	A11010

Table SM4. Antibodies used for western blot analysis.

Antígen	Dilution	Commertial Company	Catalog
**PTEN**	1:1000	Cell Signaling technology	9188
**P53**	1:1000	Leica	VP-P956
**β-Actin**	1:5.000	Santa Cruz Biotechnology	sc-1616
**GAPDH**	1:20.000	Abcam	8245
**HIS-TAG**	1:1000	Cell Signaling technology	2365
**Rabbit IgG-HRP**	1:10.000	Jackson	111-035-003
**Rabbit IgG-HRP**	1:10.000	Jackson	115-035-003

Table SM5. Primers and PCR conditions for Amplicon-NGS sequencing. DNA fragments flanking the target sequence of the RNPs targeting the indicated genes were amplified with the indicated primers and PCR conditions in 50µl PCR reactions using Taq polymserase (Biotools).

Targeted gene		Primer	PCR Protocol		
			T (ºC)	Time	Cycles
Pten	Fwd<br>Rev	TTATCTTTTTACCACAGTTGCAC<br>GTGGTTGTATCCACTTAGTGTA	95 ºC	2’	1
			95 ºC	30’’	45
			55 ºC	30’’	
			72 ºC	1’20’’	
			72 ºC	7’	1
p53	Fwd<br>Rev	CCATGCTAAGCAAGTGTTGG<br>CCCTAAGCCCAAGAGGAAAC	95 ºC	2’	1
			95 ºC	30’’	45
			55 ºC	30’’	
			72 ºC	1’20’’	
			72 ºC	7’	1

Figure SM1. Images of the Coomassie blue stained acrylamide gel showing Purified TAT-Cre band. F-T; Amicon® (flow-through) eluted volume, LB OEI Medium; Lysogeny Broth Overnight Express™ Instant medium. kDa; kilodaltons. MW: BenchMark™ protein marker.

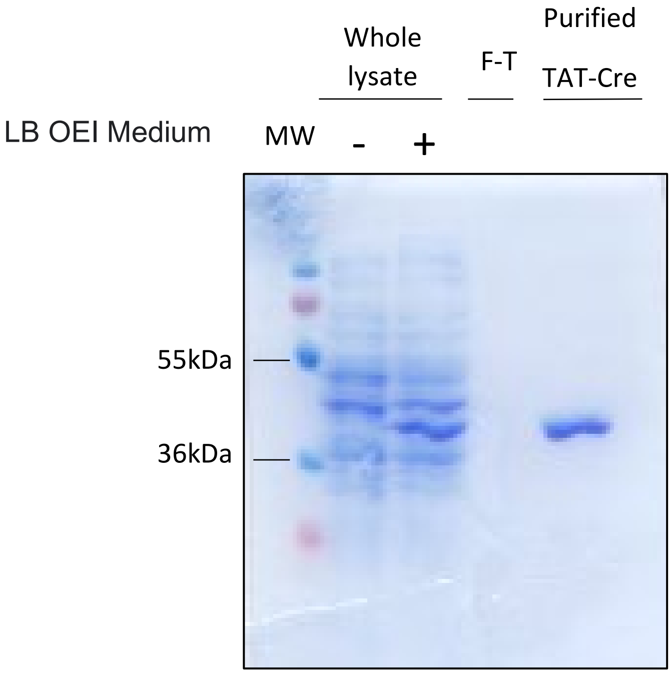

Supplementary Figures.

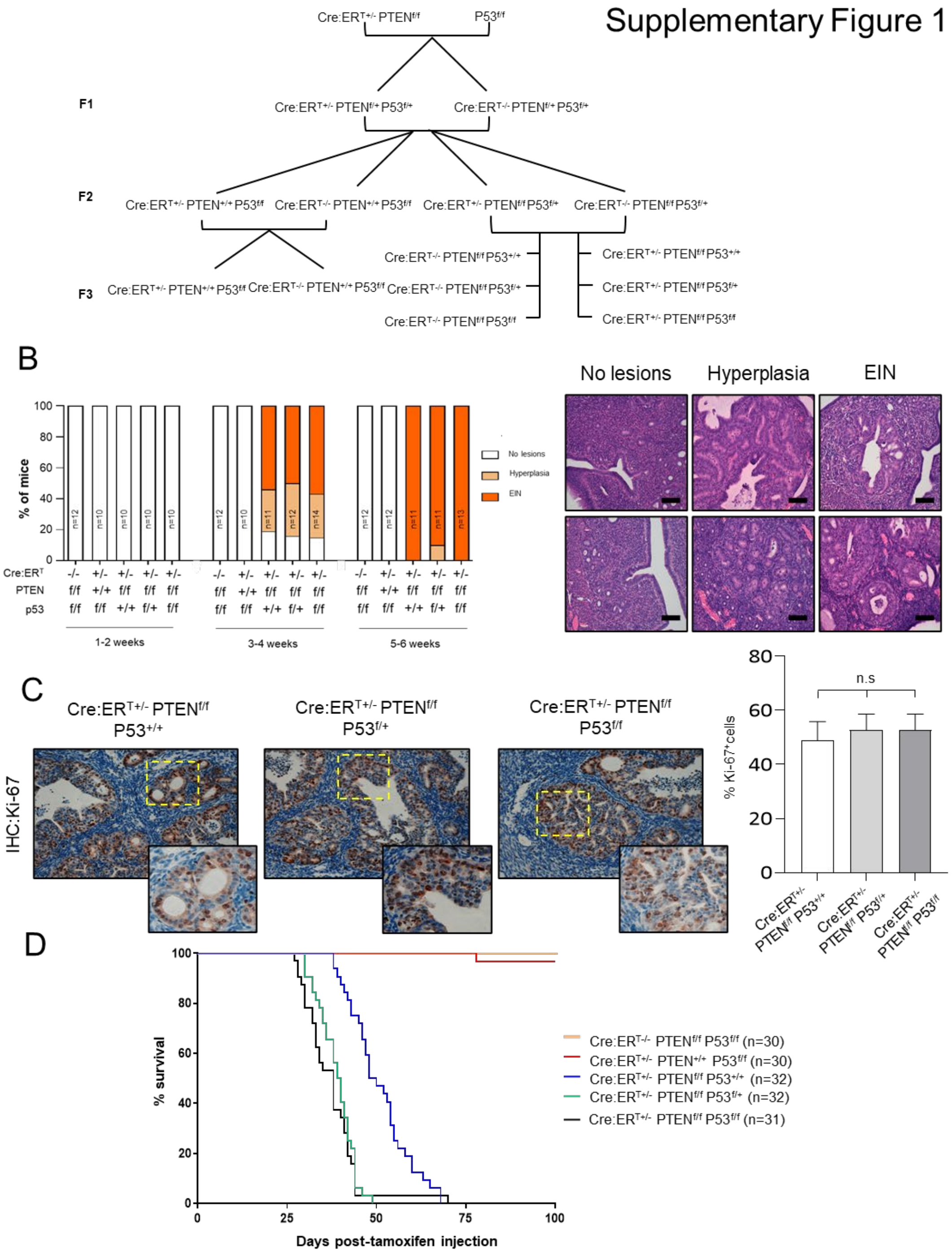

Supplementary Figure 1. In vivo Tamoxifen-inducible deletion of PTEN and P53 leads to the development of hyperplasia and non-invasive intraepithelial neoplasia. (A) Breeding protocol for the generation of murine models Cre:ERT+/-; PTENf/f; P53f/f (dKO), Cre:ERT+/-; PTENf/f; P53+/+ (PTENKO) and Cre:ERT+/-; PTEN+/+; P53f/f (p53KO). (B) Quantification and representative hematoxylin-eosin images of endometrial lesions for the indicated groups of mice. ****p<0.0001, according to the Xi^2 test, followed by Fisher's exact test. EIN (Endometrial Intraepithelial Neoplasia). (C) Representative Ki-67 immunohistochemistry images of $Pten^{+/+}$; $Cre{:}ER^{T+/-}$ $Pten^{+/+}$ $p53^{f/f}$, $Cre{:}ER^{T+/-}$ $Pten^{f/f}$ $p53^{+/+}$, and $Cre{:}ER^{T+/-}$ $Pten^{f/f}$ $p53^{f/f}$ uterine sections 6 weeks after tamoxifen injection. (D) Kaplan-Meier plot showing survival of animals $Cre{:}ERT^{-/-}$; $PTEN^{f/f}$; $P53^{f/f}$ (WT), $Cre{:}ERT^{+/-}$; $PTEN^{+/+}$; $P53^{f/f}$(P53KO), $Cre{:}ERT^{+/-}$; $PTEN^{f/f}$; $P53^{+/+}$ (PTENKO), $Cre{:}ERT^{+/-}$; $PTEN^{f/f}$; $P53^{f/+}$ and $Cre{:}ERT^{+/-}$; $PTEN^{f/f}$; P53/f(dKO). p-value <0.01. Statistics performed with the Log-rank test (Mantel-Cox).

Supplementary Figure 2

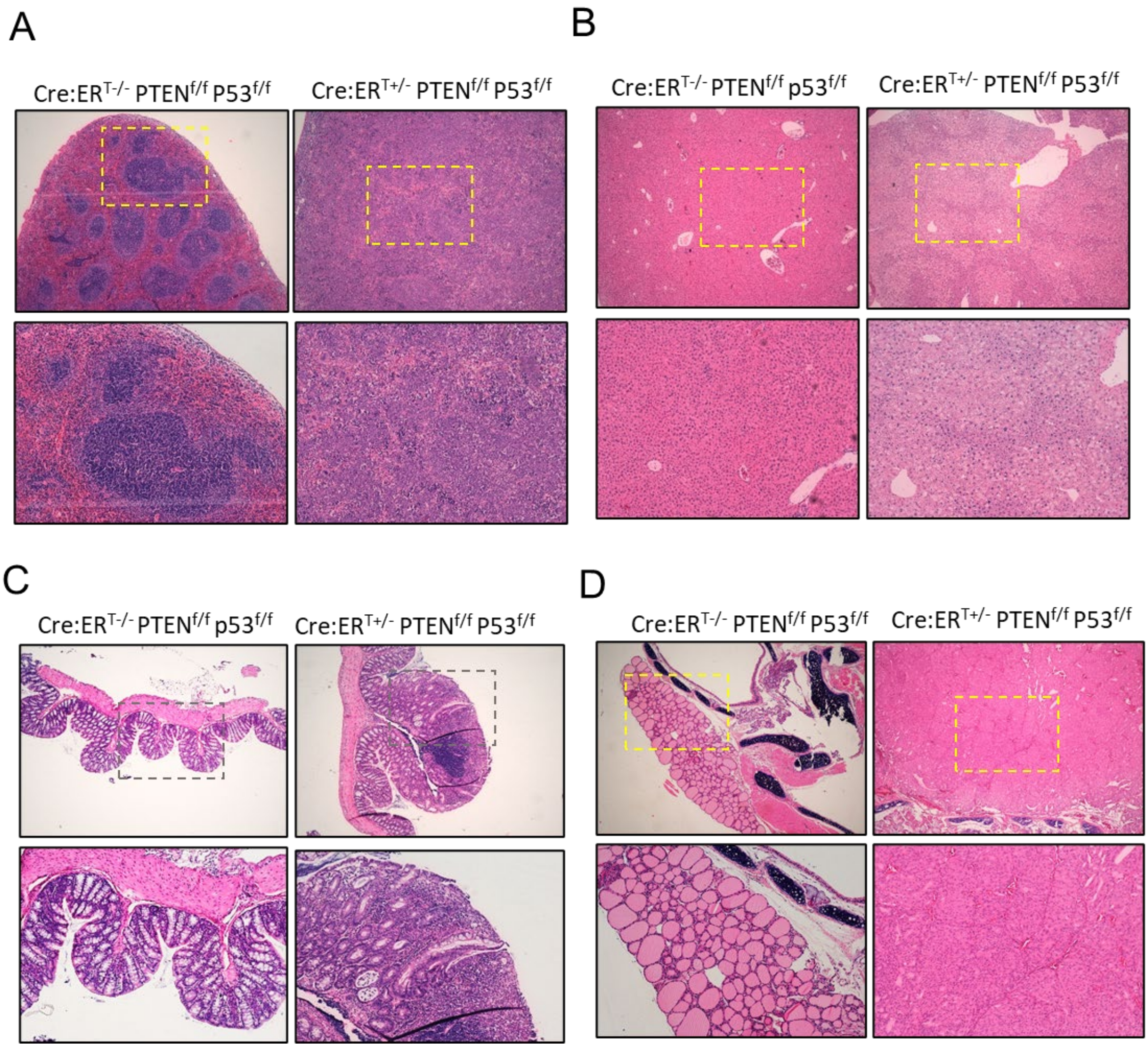

Supplementary Figure 2. Histopathological study of lesions observed in double Pten/p53 knock-out mice. Representative images of lymphoma (A), hepatocellular displasia (B), colon adenomas (C), or severe thyroid hyperplasia (D).

Supplementary Figure 3

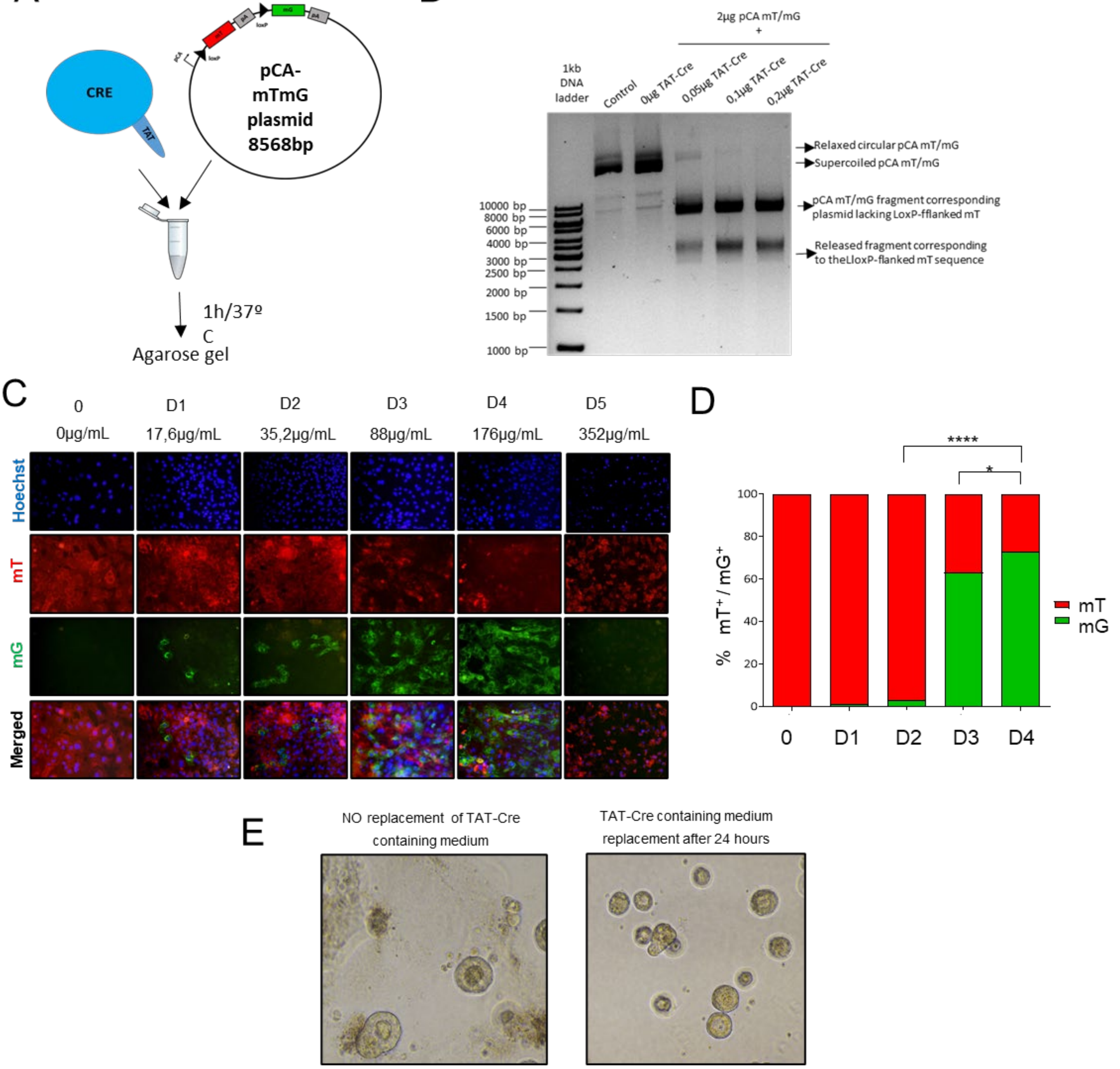

Supplementary Figure 3. TAT-Cre exhibits recombinase activity in vitro. (A) Scheme of the in vitro assay for the analysis of TAT-Cre recombinase activity using the pCA-mTmG plasmid. (B) Representative image of an agarose gel showing the DNA fragments of pCA-mT/mG plasmid generated by TAT-Cre activity. (C) Representative images of tdTomato positive (mT^{+}) or GFP positive (mG^{+}) fibroblasts isolated from $mT/mG^{f/f}$ mouse ears 4 days after being treated with the following TAT-Cre concentrations: 0µg/mL (0), 17.6µg/mL (D1), 35.2µg/mL (D2), 88µg/mL (D3), 176µg/mL (D4) and 352µg/mL (D5). Images captures at 20X. (D) Quantification of tdTomato positive (mT^{+}) or GFP positive (mG^{+}) fibroblasts isolated from $mT/mG^{f/f}$ mouse ears 4 days after being treated with the following TAT-Cre concentrations: 0µg/mL (0), 17.6µg/mL (D1), 35.2µg/mL (D2), 88µg/mL (D3), 176µg/mL (D4). D5 quantification was omitted due to the lack of green cells caused by TAT-Cre cytotoxicity. Data from n=3 independent experiments. *p<0.5 ****p<0.0001, using a one-way ANOVA analysis, followed by a Bonferroni multiple comparison test. (E) Representative images of endometrial organoid cultures treated with 352µg/mL TAT-Cre the day of plating. Cell culture medium containing TAT-Cre was replaced 24 hours after (right image) or left in contact with cells. Images were taken 10 days after cell plating.

Supplementary Figure 4

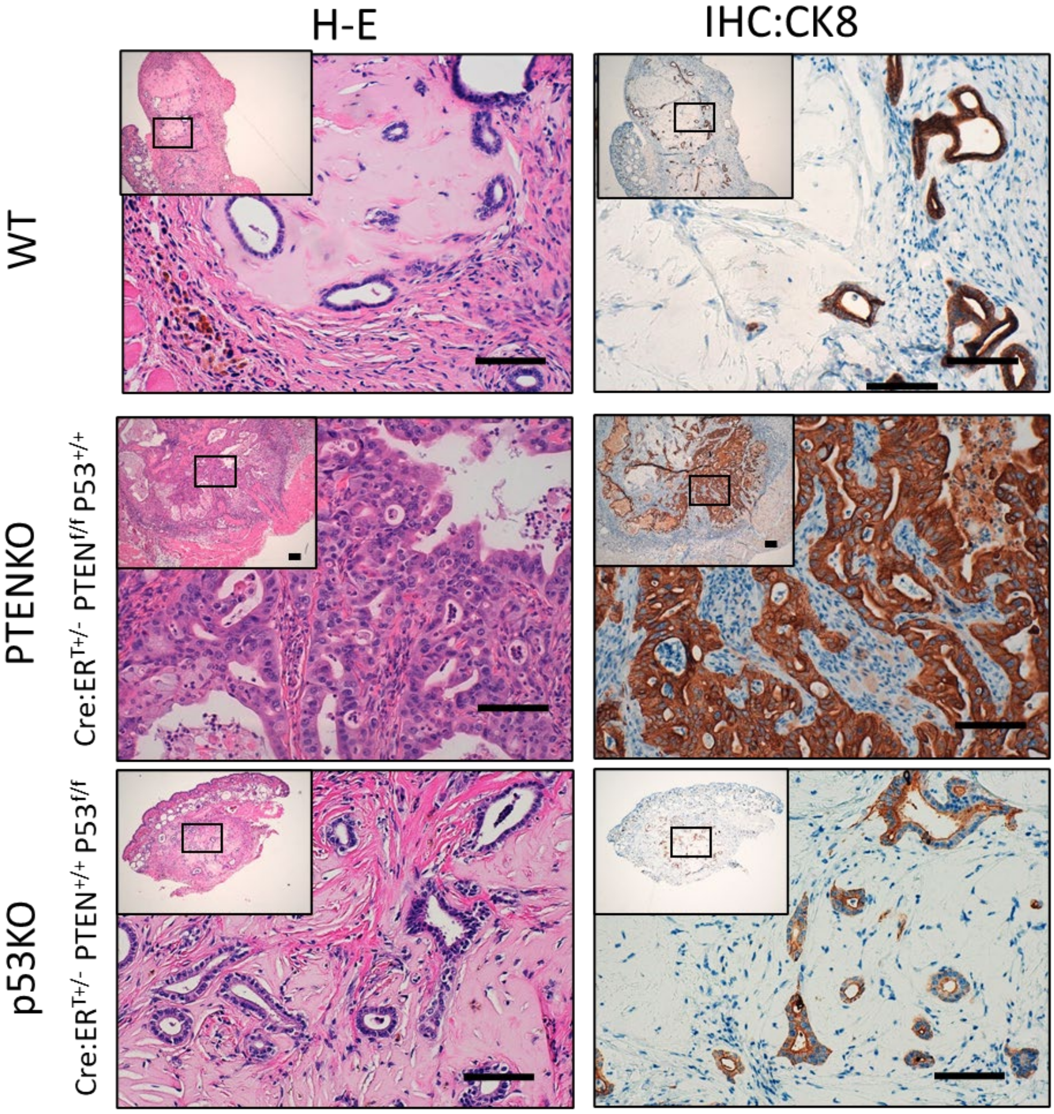

Supplementary Figure 4. Histopathology of WT, PTENKO or p53KO xenotransplants.. Representative images of hematoxylin-eosin images (H-E) and cytokeratime-8 (CK8) immunohistochemistry on endometrial tissues and lesions developed from xenotransplated endometrial epitelial cells of the indicated genotypes.

Supplementary Figure 5

	TAT-Cre	CRISPR/Cas9	Traditional breeding of Cre mice with conditional floxed mice
Mice requirements	Floxed mice	No requirement	Floxed mice + Cre expressing mice
Efficiency	+++	++	Highly variable depending on Cre expression
Targetable genes	Floxed genes	Any gene	Floxed genes
Equipment requirements	No required	In vivo electroporator	No required
Specificity for endometrial epithelial cells	+++	+++	+/-
Age of mice at time of delivery and inducibility	Any/inducible	Any/inducible	Variable, depending on availability of inducible Cre expressing mice
Multiplexing	+ (Requires breeding of multiple floxed mice, time consuming)	++++	+/- (Requires breeding of multiple floxed mice and Cre expressing mice, time consuming)
Flexibility	++	++++	+

Supplementary Figure 5. Chart table comparing the properties of different available systems for genetic deletion of genes of interest in mouse endometrium.
